# P-Wave detection using deep learning in time and frequency domain for imbalanced dataset

**DOI:** 10.1016/j.heliyon.2021.e08605

**Published:** 2021-12-14

**Authors:** Rhesa Aditya Sugondo, Carmadi Machbub

**Affiliations:** a School of Electrical Engineering and Informatics, Institut Teknologi Bandung, Bandung, West Java, Indonesia; b Center for Earthquake Science and Technology, Research Center for Disaster Mitigation, Bandung, West Java, Indonesia

**Keywords:** AFAP, Deep learning, Frequency domain, SMOTE

## Abstract

Early tsunami and earthquake warning systems need a good Automatic First Arrival Picking (AFAP) subsystem to determine the earthquake arrival time. This subsystem has a time-domain earthquake signal as the input and the arrival time of the primary earthquake wave (P-Wave) as the output. There are several methods of AFAP that are widely used nowadays, one of them is Short Term Average/Long Term Average (STA/LTA) fused with the AR-AIC method. Even though this method is real-time, its performance is still relatively low. With similar characteristics between the seismic signals and image data, utilising deep learning on AFAP can further increase its performance. The seismogram channels can be seen as the image height, and the signal at a certain window can be seen as the image width. Unfortunately, these image data will be considered an imbalanced dataset. In this research, deep learning with time domain and frequency domain are proposed as inputs with the Synthetic Minority Oversampling Technique (SMOTE) method. Deep learning is used because of its ability to generalise well on a huge dataset, while SMOTE is used to overcome the imbalanced dataset problem. With this proposed system, the accuracy is 99.3%, the Root Mean Square Error (RMSE) is 0.202 seconds, and the maximum execution time is 0.17 seconds with a periodic time of 0.4 seconds. With these results, the AFAP system has good results for estimating the first arrival earthquake time.

## Highlights

• Convolutional neural networks with time and frequency domain can detect Earthquake Primary Wave (P-Wave) signals well.
• The Synthetic Minority Oversampling Technique (SMOTE) can increase the imbalanced earthquake dataset training performances.
• P-Wave detection with several time windows and classifiers can increase overall performance, surpassing traditional method.
• The proposed system can achieve a 0.17-second execution time with 0.4 seconds periodic time.

## Graphical abstract

**Figure:**
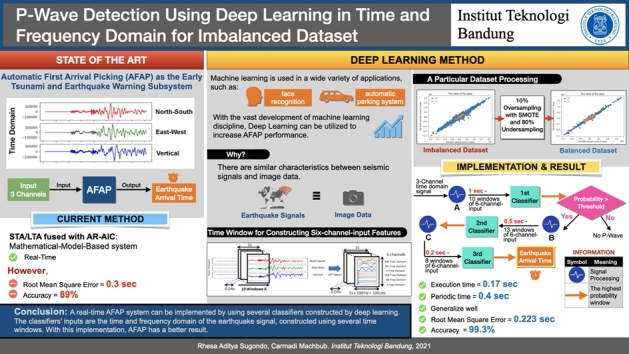

## 1 Introduction

An earthquake is a disastrous phenomenon that has a hazardous impact on the environment. One of the popular systems to minimise this effect is an early earthquake and tsunami warning system. This system consists of several subsystems. One of these is Automatic First Arrival Picking (AFAP), which estimates the arrival time of an earthquake signal [1]. The arrival time of an earthquake signal is crucial for calculating the earthquake depth. This earthquake depth is one of the important parameters to describe its potential to trigger a tsunami [2].

The earth's movement can be measured through a seismograph. The measured signal from the seismograph is a three-channel acceleration of the earth's movement versus time. An earthquake signal consists of a Primary Wave (P-Wave), Secondary Wave (S-Wave), and a Surface Wave for each channel. As the name implies, the first captured earthquake wave will be the Primary Wave [3].

Earthquake and tsunami early warning systems need to know the first time the earthquake signal arrived [1]. The information on this first arrival is encapsulated by the time the P-Wave appears. The usual method for detecting the P-Wave at the majority of the seismic tower is by using a Short Term Average/Long Term Average (STA/LTA) and Auto-Regressive Coefficient (AR-AIC) method [4]. The accuracy of this method to distinguish earthquake signals and noise signals is relatively low, 89%, especially when the signal has a low SNR [5]. It has a 0.3-second root mean square error (RMSE) between the estimated and the actual arrival time [5]. In the end, most seismologists have to double-check the results from the AFAP.

With the development of the machine learning method, dataset, and computational power, there is a considerable chance that AFAP can increase its accuracy. Deep learning is one of the machine learning disciplines often implemented in speech and image recognition with the Convolutional Neural Network (CNN) architecture [6]. CNN has been widely used in image recognition problems because it can compute faster when overcoming a large dataset and will have less risk of overfitting relative to the other architecture.

In the image recognition discipline, the input will usually be the red, green, and blue pixel values from the input image. This research tries to bring the principle of image recognition to the AFAP system for earthquake and tsunami warning systems. As mentioned above, the earthquake signal also consists of three channels, which are the north-south channel, west-east channel, and vertical channel [7]. The considerable size of the earthquake dataset is another reason for using the deep learning method. Another reason is that the earthquake phenomenon has not yet been modelled perfectly. The deep learning architecture can extract the valuable new pattern of the earthquake signal and get a better understanding. There has been similar research about this [7], and with the Wenchuan earthquake dataset, the accuracy of distinguishing between the noise and earthquake signals is above 95%.

This research will use a more general and different dataset but with the same sampling frequency. There will be a change in the network architecture, such as the number and the type of layers [8]. Then, the proposed system will include a subsystem in one of the micro-seismic mining systems [9], which has included the frequency domain of the signal as a feature of the neural network. Also there will be additional pre-processing data, a dataset balancing method, the SMOTE, to further improve the performance. Thus, this research proposes a CNN with six features as the inputs. These features are the three time-domain channels and the three frequency-domain channels.

With the pre-processing data, additional features, and changes in the architecture, this research aims to create an AFAP system with an accuracy of 95% and RMSE lower than 0.3 seconds. These numbers were chosen to fix the STA/LTA and AR-AIC performance qualities.

## 2 Related works

The history table of several related works for determining the arrival time of an earthquake is shown in Table 1.

**Table 1:** Related works.

	Method	Performance
STA/LTA picker	Calculates the characteristic function, which is a fraction of a short-term window and a long-term window. If this fraction reaches a certain threshold, then it would be a P-Wave [4].	RMSE at 0.3 seconds Accuracy at 89% [5].
AIC picker	Assumes that intervals before and after P-phase arrival are two different stationary processes. This method has to minimise the AIC function to determine when the P-Phase comes [10].	At a similar level with STA/LTA, error at 0.3 seconds and accuracy at 89% [5].
EMD-based picker	Decomposes a signal adaptively. Then the P-Phase onset is determined by the selected main IMFs that retain P-Phase arrivals well [10].	Does not work well on a low SNR environment [10].
ANN Identification	Uses spectrogram representation as a feature of the Artificial Neural Network [11].	Execution time more than 3 seconds [11].
XTF-CNN	Uses frequency and time domain series as the features of the Convolutional Neural Network. This method also has a subsystem to map it back as explainable traces [9].	Accuracy at 95.17% [9].
CNN	Uses a Convolutional Neural Network with a drop-out layer [8].	Accuracy at 85% [8].
CPIC	Uses a CNN with Phase Detector and Phase Picker architecture to determine the P-Wave arrival time [7].	RMSE below 0.2 seconds and Accuracy is 96% [7].

The first three methods are the traditional method for determining the arrival time of the earthquake, and because of their real-time properties, they are still used on most seismic towers now. The last four methods are some of the approaches using neural network architecture that have recently been researched. One of the methods used as a role model is the CNN-based Phase-Identification Classifier (CPIC).

## 3 Dataset and pre-processing

### 3.1 Dataset and moving window

The dataset for this neural network is the Standard for the Exchange of Earthquake Data (SEED). This SEED format is widely used to represent stream seismograph data. The SEED format consists of the part containing time-series data for several channels, called the mini-SEED, and some metadata from that station, called data-less SEED [12]. For this research, the mini-SEED will be considered as a stream object, being the dataset format used.

The source of the mini-SEED data in this research is the Incorporated Research Institutions for Seismology (IRIS) website [13] and from the STEAD (Standford Earthquake Dataset) [14]. Mainly, this research uses STEAD data as training data. The data selection criteria are seismic events from the year 2000 that have a magnitude over M5.0. Also, the mini-SEED data will consist of three-channel seismographs of the nearest station from the epicentre of the event. We will also use data that have been sampled at 100 Hz by the seismographs. The raw mini-SEED data label is the arrival time picked by several picking methods from STEAD when the P-Wave first arrives at that event. Most of the picking methods are manual picking by the seismologist.

This research uses the ObSpy and NumPy libraries to construct the input for the neural network. With these libraries, the mini-SEED data will be processed into a stream object. Inside this object, there are several trace objects. Each trace represents the seismograph on a particular channel and station. This trace object consists of several important attributes, such as time-series data inside the data attribute, some sampling, and time information inside the status attribute. We also had to access the time when the P-Wave first arrived.

After accessing the time-series data, there will be some processes with a moving window to construct the data. This research uses a 100 Hz dataset. The proposed system will have three kinds of moving windows.

1. Moving Window A: Length of 1 second for the 1^st^ phase detector subsystem
2. Moving Window B: Length of 0.5 seconds for the 2^nd^ phase detector subsystem
3. Moving Window C: Length of 0.2 seconds for the phase picker subsystem

The moving window A will try to detect P-Wave on a 1-second window for every 0.04 seconds. If the P-Wave is detected on that window, the system will use the moving window B to get a more detailed time from the moving window A. This moving window B has a length of 0.5 seconds and will detect the event every 0.04 seconds. These processes are repeated until the system reaches the moving window C, which has the shortest time window. This means that step by step, the picking time will be more detailed.

**Figure 1:**
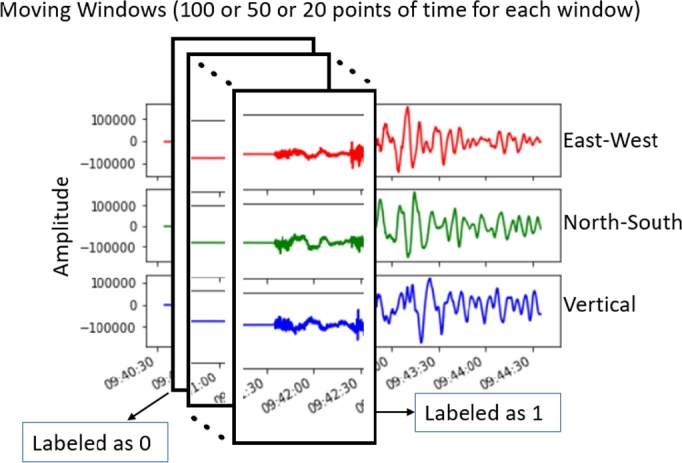
Moving windows method to capture several periods of earthquake.

With a 100 Hz sampling rate, moving window A creates 100 sample points from the signal. The moving window B will create 50 sample points from the chosen signal from time window A. The moving window C will create 20 sample points from the chosen signal from moving window B. If the arrival time of the P-Wave is included inside a particular moving window, then that window will be labelled “1”, and otherwise, it will be “0”, as shown in Fig. 1.

**Figure 2:**
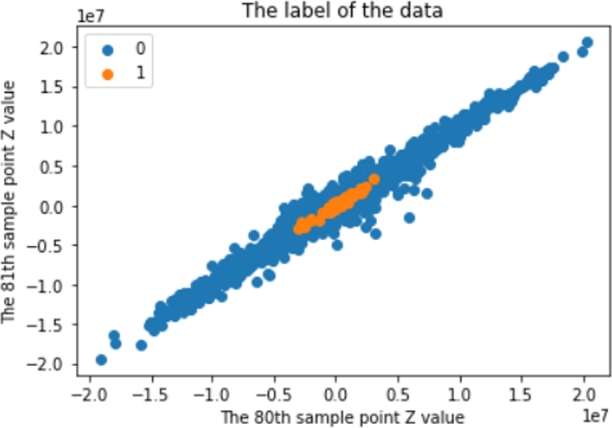
An example of signal dataset after captured by the moving windows method.

With those principles, the results from the dataset cooking are shown in Fig. 2. This particular data-cooking process picked 2,278 earthquake events with the criteria described above.

### 3.2 Discrete cosine transform

After a standard filtering process to increase SNR, the signal will be transformed to the frequency domain for classifier input features. The discrete cosine transform is chosen because this system only needs the real part of the signal [9]. Formula (1) was used for this research implementation.

(1) $$F(k)=c(k)\sum_{x=0}^{N-1}f(x)\cos(x+\frac{\pi k}{2N})$$

**Figure 3:**
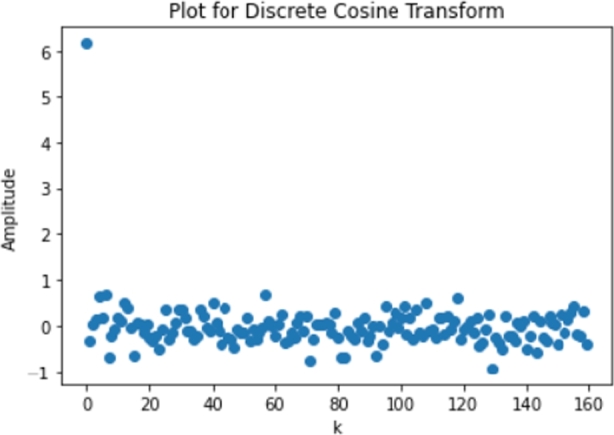
Frequency domain of one channel of the signal.

One of the results of the transformation is shown in Fig. 3. Note that the amount of sampled signal in the time domain will be equal to the amount of sampled signal in the frequency domain by using this discrete cosine transform. This means that the dimensions for the time domain and frequency domain will be equivalent.

### 3.3 Balancing the dataset

The proportion of P-Wave events and non-P-Wave events is imbalanced. In a one-minute earthquake event, there will be around 100 moving windows consisting of P-Wave and around 5,900 consisting of non-P-Wave events. Because of this, the training process of the system needs a more balanced training pattern for calculating the best result. One way to overcome this problem is by mining a large amount of data from earthquake events and under-sampling the non-P-Wave event. If the amount of data is not sufficient, another way is by undersampling the non-P-Wave event and oversampling the P-Wave event. In this research, the system will perform oversampling and undersampling of the data due to the relatively small amount of data.

To implement this method, the training system uses SMOTE, which will make a new P-Wave event based on the pattern from the dataset [15]. This research will use the SMOTE with a 10% sampling strategy and undersampling with a 70% sampling strategy from the total dataset that we have processed before. These numbers were chosen because there is a limitation of the training process RAM. The training data size target is 4 GB, so the remaining RAM space can be used by another process. With this strategy, the huge imbalanced dataset will be reduced to a more compact and balanced dataset. Notice that there is a large process of undersampling. This strategy can be done only because there are so many data repetitions in the majority class, the non-P-Wave class.

**Figure 4:**
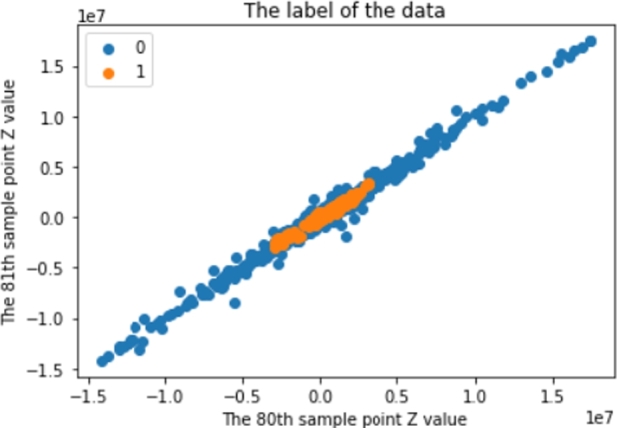
An example of signal dataset after the balancing process.

Before using the SMOTE with those strategies, Fig. 2 shows that there are 227,800 P-Wave class data and 13,440,200 non-P-Wave class data. After using those strategies, Fig. 4 shows the results of implementing the SMOTE. There are around 375,000 P-Wave class data and around 375,000 non-P-Wave class data.

## 4 Method and experiment

### 4.1 Specifications

The purpose of this research is to develop a system that works better than the traditional method. The traditional methods are the STA/LTA and AR-AIC methods. Hopefully, this new AFAP system can better support an early warning system. Because of that, there are several specifications for the AFAP system that will be designed, as shown in Table 2.

**Table 2:** Specification.

Parameter	Criteria
RMSE	Lower than 0.3 seconds
Precision	Higher than 95%

### 4.2 System architecture

**Figure 5:**
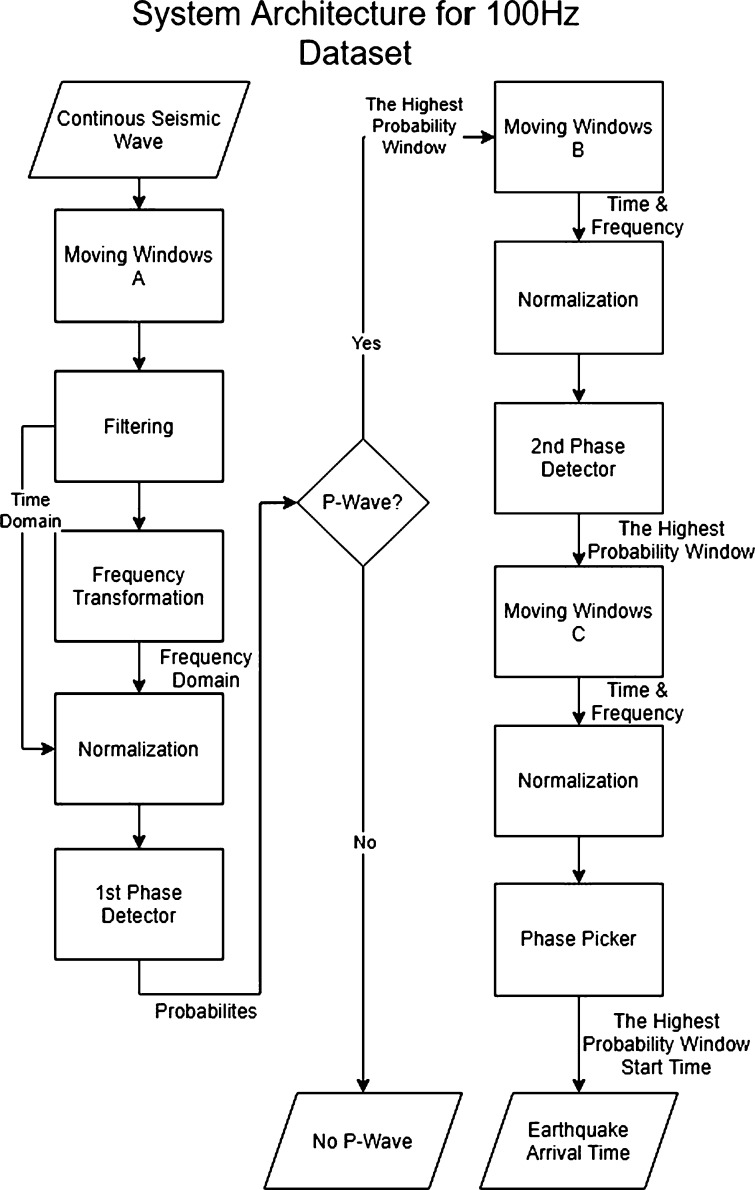
Proposed system architecture.

The system itself consists of several subsystems. Generally, this system will receive a continuous seismic wave and will produce an output of the first time the P-Wave arrived as soon as the system detected it. This system is constructed based on the main principle of the CPIC research system [7]. The system architecture is shown in Fig. 5 and explained in Table 3, Table 4.

**Table 3:** Subsystems (1).

	Input	Process	Output
Moving Windows A	The stream data of seismic waves.	Constructing input with time windows.	Several windows that consist of some sample points.
Moving Windows B and C	The window with the highest probability of P-Wave.	Constructing input with smaller time windows.	Several new windows that consist of some sample points.
Filtering	Several windows from the signal.	Filtering to distinguish noise signal and seismic signal.	Several windows of the filtered signal.
Frequency Transformation	Several windows of the filtered signal.	Discrete Cosine Transformation [9].	Discrete Frequency domain of the filtered signal.
Normalisation	Several windows that consist of time and frequency domain.	Scaling process.	Several normalised windows.
1^st^ Phase Detector	Several windows that consist of time and frequency domain.	Classify those windows to a P-Wave event or a non-P-Wave event [8].	Probabilities of P-Wave event for those windows.

**Table 4:** Subsystems (2).

	Input	Process	Output
2^nd^ Phase Detector	Several windows that consist of time and frequency domain.	Classify those windows to a P-Wave event or a non-P-Wave event.	The window with the highest probability of P-Wave.
Phase Picker	Several windows that consist of time and frequency domain.	Classify those windows to a P-Wave event or a non-P-Wave event.	The start time of the highest probability window (P-Wave first arrival time).

There are many differences between the proposed system and the CPIC, such as the time window principle, the time window size, the amount of phase detector subsystems, the input features for the classifier, the balancing method for pre-processing the data, the dataset used to train the classifier, the architecture of the classifier, and the classifier output.

One of the reasons the proposed system differs from the CPIC system is because the CPIC used 11 convolution layers with a single-phase detector. Thus, it is slow in training and requires huge resources. The proposed system only uses two layers of a convolution network, with the trade-off increasing the number of phase detectors to two.

CPIC used only the time domain as an input. The proposed system aims to increase the performance of the system by utilising another point of view. Thus, the proposed system adds the frequency domain as an input. The proposed system also wants to increase training performance more by using the balancing dataset method, which is not used at CPIC.

In addition, CPIC used a 2,000 sample time window for both phase detector and phase picker, which is hard to implement as a real-time system. Thus, the proposed system uses a 100-sample time window and will decrease as the system wants to get the detailed time. This difference in the time window principle will make the system relatively more applicable as a real-time system than the CPIC. The trade-off using smaller time windows is low classifier performance because the input for the classifier will be smaller. However, with the help of frequency domain and dataset balancing, the system performance will still be in a range of specifications.

There are also many algorithms designed to maximise accuracy and minimise errors, that are not used at CPIC. In short, the similarity with the CPIC is the principles of utilising time windows for picking and using more than one classifier.

The first AFAP process occurs at the first phase detector. In each step, the system processes the probability from the first classifier in a 10-time-windows. Thus, the total length is 0.4 seconds. If the probability of the P-Wave signal is higher than a certain threshold and pops up more than several times, it means the classifier is sure that the detected event is a P-Wave signal. However, if it only pops up below six times, then it will be considered noise. This is because several noises resemble earthquake signals, which is why we need a voting system from several closed-position time windows to make sure.

If there is an earthquake signal, the overall system needs to grind this result in a more detailed time. The grinding process needs to be done step by step by using several phase detectors with shorter time windows, until it reaches the phase picker so that the overall system can maintain the RMSE well.

### 4.3 Classifier architecture

**Figure 6:**
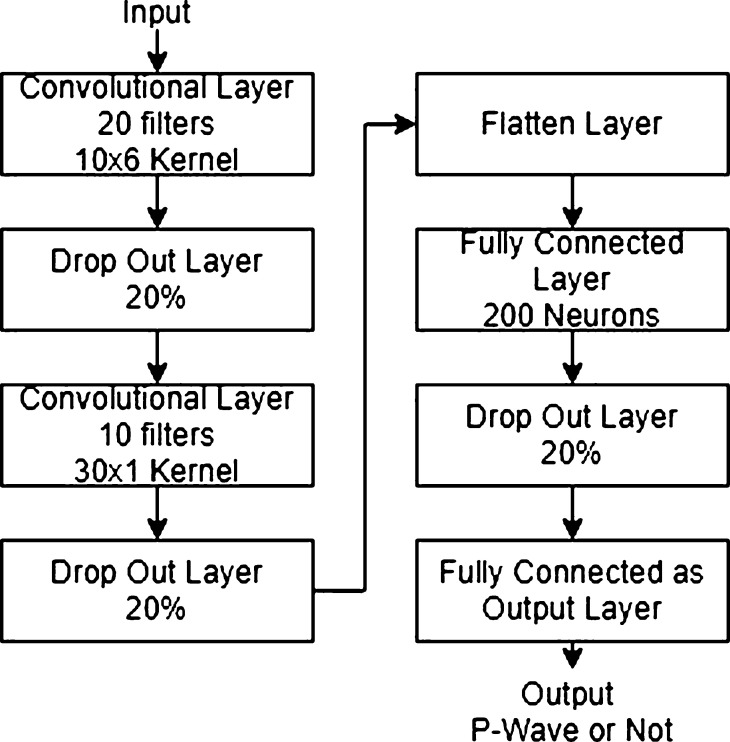
Proposed classifier architecture.

It is interesting to look at the data as an image because of its three-time-domain channels and three-frequency-domain channels. As an image, these channels can be seen as the image width and the time sample as the image length. Because of that, this system uses a CNN with a two-dimensional kernel and a drop-out layer. Fig. 6 shows the architecture of the phase picker and phase detectors, based on previous research, with several changes [8]. The changes themselves consist of the input dimension (because in this research, we use three-time-domain channels and three-frequency-domain channels) and the zero paddings to maintain the dimension. By using zero paddings, the classifier performance will be better, but it will have more parameters. For this four-hidden-layer classifier system, a large number of parameters will not disturb the overall performance too much.

The first layer is a 2-D convolution layer with 20 kernels. Each kernel has a size of 10 x 6 and has a rectified linear unit as an activation function. The kernel size derives from its 6-channel input. Next is the drop-out layer, with a probability of 20%. The third layer is a 2-D convolution layer with ten kernels. Each kernel has a size of 30 x 1 and also has a rectified linear unit as an activation function. The fourth layer is the drop-out layer, with a probability of 20%. With these configurations, the feature will be deeper and condensed to a smaller size. After these processes, there will be a flattened layer whose output can be fed to dense layers to increase accuracy.

### 4.4 Classifier set-up

After the raw dataset was processed to trace the data, it was processed by the moving window. After the array processing, the dataset was split into training and test data. The training system uses stratified shuffle split to maintain the proportion of the P-Wave event at 20% for test and 80% for training. Thus, for the training process, the P-Wave class consisted of around 300,000 data and the non-P-Wave class also consisted of around 300,000 data. For the testing process, the P-Wave class consisted of around 75,000 data and the non-P-Wave class consisted of around 75,000 data.

**Figure 7:**
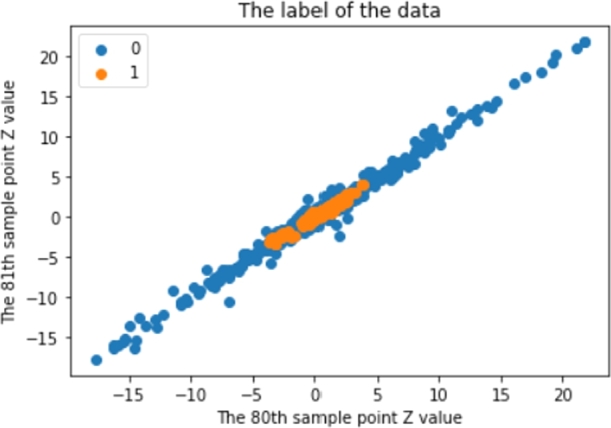
An example of a normalised dataset.

After splitting the data, is feature scaling for the 3-Dimension dataset that has been set up. The training system uses a standard scaler with the pivot in the first dimension. Thus, the amplitude values were converted down, as shown in Fig. 7.

### 4.5 Classifier training

After several set-ups, the training system trained three different classifiers, but with the same architecture. The difference was in the input size related to the respective moving window for that subsystem. Detector Classifiers have an input of 100 features from moving window A, 50 features from moving window B, and the Picker Classifier has 20 features as input from moving window C.

Training set-up for classifiers uses the cost function of binary cross-entropy, Adam optimiser, early stopping at ten iterations, and validation split by 20%. The training metrics focus on loss and accuracy.

#### 4.5.1 1^st^ phase Detector Classifier

**Figure 8:**
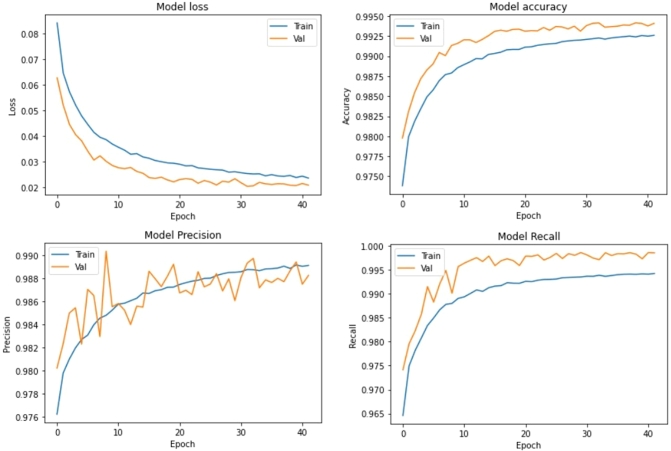
1^st^ phase Detector Classifier performance.

The results history for the 1^st^ Detector Classifier is shown in Fig. 8.

For this 100-feature input, this training system obtained 99.33% accuracy for both training and validation. When using the test dataset, the result was accuracy at 99.3% and loss at 0.0242. Consequently, the classifier model is not overfitting because the accuracy from training and test are relatively similar. Note that this was the result from the 1^st^ phase detector, which has the biggest time window. Therefore, it is natural that the accuracy is great because of 100 features as an input. This means that if this subsystem detects a P-Wave signal, it can not distinguish the time below 1 second (100 features). The results from this subsystem are on 1 second delta time, so it is not yet detailed and accurate. This overall system needs to grind this result at a more detailed time. The grinding process needs to be done step by step so that the overall system can maintain the RMSE well. The 1^st^ phase detector accuracy can be assumed as the overall system accuracy because the rest of the subsystems will not work when this 1^st^ phase detector doesn't detect the P-Wave. That is why this system's accuracy is 99% with the threshold at 0.96.

Fig. 8 shows that the precision of phase detector 1 is good. The precision value is in the range of 98.87% at the last epoch for both validation and training. It means that the 1^st^ phase detector can overcome the false positives well according to the formula for precision shown in Equation (2).

(2) $$Precision=\frac{TruePositives}{TruePositives+FalsePositives}$$

With lower false positives compared to true positives, the 1^st^ phase detector can classify the P-signal well relative to the false alarm phenomenon. This false alarm is defined as a no-P-signal but classified as a P-signal (false positive).

Fig. 8 shows that the recall from the 1^st^ phase detector is classified as good. The recall value is in the range of 99.63% in the last epoch for both validation and training. The formula for recall is shown in Equation (3).

(3) $$Recall=\frac{TruePositives}{TruePositives+FalseNegatives}$$

A higher recall value indicates that the false-negative value is relatively smaller than the true-positive value. A false-negative event occurs when a signal is categorised as noise while it should be a P-signal category. In other words, a large number of false negatives indicates that the system is less sensitive in detecting P-signals. With a lower number of false-negative values, it can be said that the 1^st^ phase detector is sensitive in detecting P-signals.

With a high precision and recall value, the 1^st^ phase detector can position itself precisely to detect P-signals. Phase detector 1 is sensitive to P-signals and is careful not to give a false alarm. Indeed, this is the desired behaviour.

#### 4.5.2 2^nd^ phase Detector Classifier

**Figure 9:**
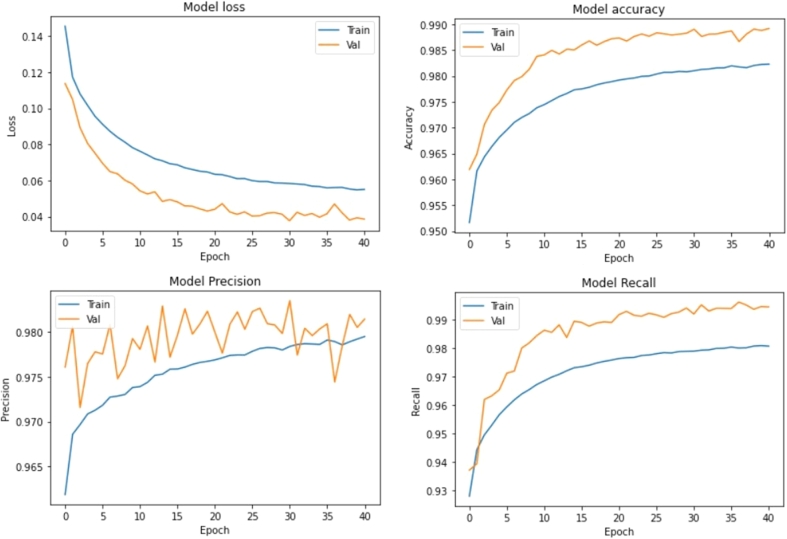
2^nd^ phase Detector Classifier performance.

The results history for the 2^nd^ Detector Classifier is shown in Fig. 9.

For this 50-feature input, this training system obtained 98.58% accuracy for both training and validation. When using the test dataset, the result was accuracy at 98.3% and loss at 0.0393. According to that result, the classifier model is not overfitting because the accuracy from training and test are relatively similar. Note that the results from the 2^nd^ phase detector show accuracy was decreasing. This is natural because the input dimension itself decreases to 50 x 6 features. This means that if this subsystem detects a P-Wave signal, it can't distinguish the time below 0.5 seconds. The results from this subsystem are on 0.5 second delta time, so it is not detailed and accurate yet, compared to our specifications. Therefore, this overall system still needs to grind this result to a more detailed time.

Fig. 9 shows that the 2^nd^ phase detector precision is good. The precision value was 98.05% at the last epoch for the validation and training process. This means that the 2^nd^ phase detector can overcome the false positive case well.

Fig. 9 shows that the 2^nd^ phase detector recall is also good. The recall value was 98.75% at the last epoch for the validation and training process. The high value of recall means that the 2^nd^ phase detector is sensitive for detecting the P-Wave signal.

With the high value of precision and recall, the 2^nd^ phase detector can detect the P-Wave signal well. The precision and recall value also decreases compared to the 1^st^ phase detector. This is similar to the accuracy value because of the smaller feature input that is fed to the 2^nd^ phase detector.

#### 4.5.3 Picker Classifier

**Figure 10:**
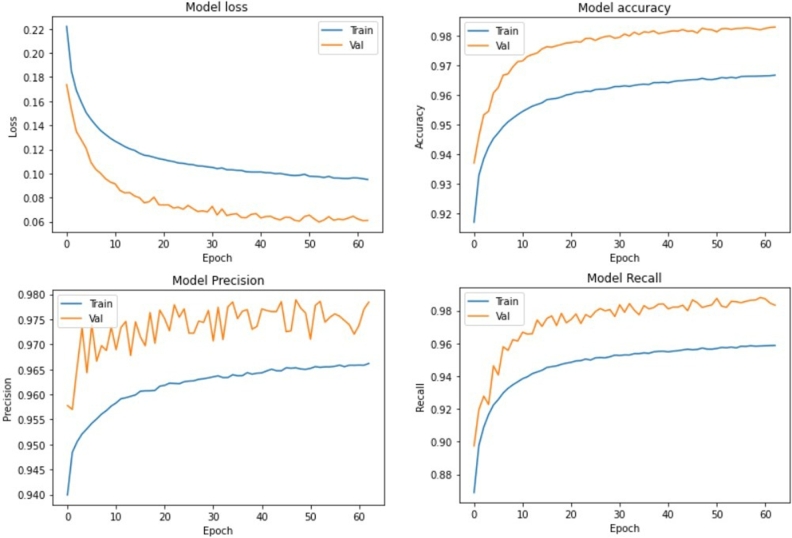
Phase Picker Classifier performance.

The results history for the Picker Classifier is shown in Fig. 10.

For 20 features input, this training system achieved 97.5% accuracy for both training and validation. When using the test dataset, the result was accuracy at 98% and loss at 0.06. These results show that the classifier model is not overfitting, because the accuracy from training and test are relatively similar. Note that we choose the picker time window to be 0.2 seconds to reach the specification of the problem. The specification is RMSE at 0.3 seconds, so it is beneficial to choose the time window lower than that. However, for the record, if a shorter time window is chosen, the features input size will decrease more and more, making the classifier result no longer valid.

As shown in Fig. 10, the phase picker precision is also good. The precision value was 97.23% at the last epoch for the validation and training process. This means that the phase picker can overcome the false alarm case well.

Fig. 10 shows that phase picker recall is also good. The recall value was 97.1% at the last epoch for the validation and training process. The high recall value means that the phase picker is still sensitive enough to detect the P-Wave signal.

With the high value of precision and recall, the phase picker can detect the P-Wave signal well. The precision and recall value also decreased compared to the 1^st^ phase detector and the 2^nd^ phase detector. This is similar to the accuracy value. This is natural because of the smaller feature input that is fed to the phase picker.

## 5 Overall system results

**Figure 11:**
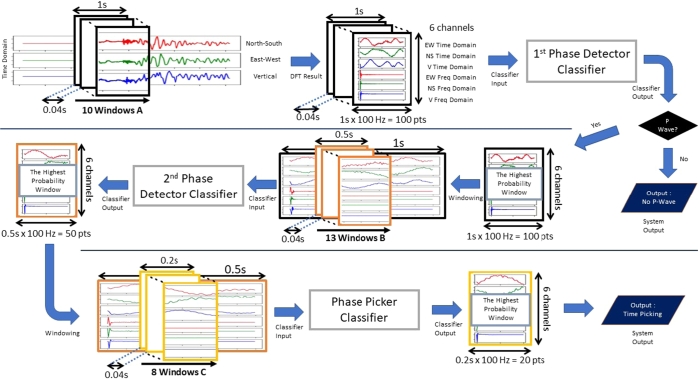
Earthquake signal processing steps.

This section consists of conducting a new seismic signal aside from that training dataset to the overall AFAP system, not just to each classifier. 2,000 continuous earthquake events were tested. The process is shown in Fig. 5. In its application, the signal that was fetched for each time step of the system is a 10-time-windows, which are 0.4 seconds. Therefore, it can be said that the periodical time for the AFAP task is 0.4 seconds. Fig. 11 shows the step-by-step system process for its application.

**Figure 12:**
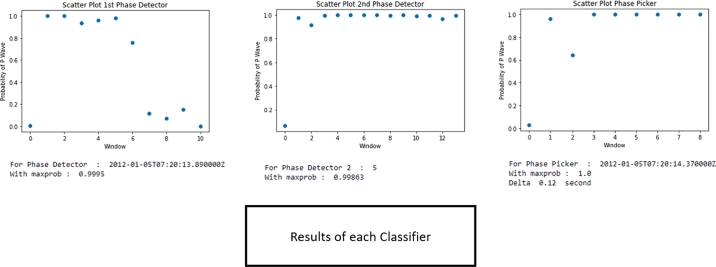
Processing earthquake step by step results.

The seismic signal is processed with a moving window, preprocessed, and fed to the neural network. First, it is fed to the 1^st^ Detector Classifier. If that window contains a P-Wave arrival event, it is fed to the 2^nd^ Detector, and then to the Picker Classifier. Fig. 12 is the step-by-step result from the systems until it can get the arrival time from one earthquake event.

The validation process of error performance uses two thousand earthquake events. 60% of this data is the training dataset, while the rest of the dataset is for validation and testing during the training process. This means that the system is validated using this 40% of new data. Note that the training process uses the stratified shuffle split method. This means that the arrangement of the data for the validation process is completely different from the training process. The arrangement of the data is a stream of data connected to one another during the validation process.

**Figure 13:**
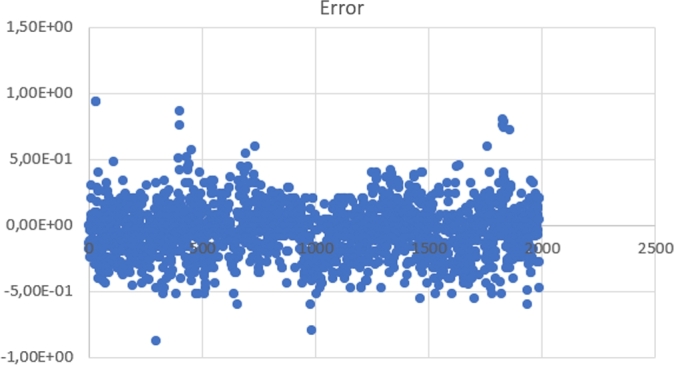
Error time for 2,000 earthquake events.

From the 2,000 earthquake events, there were 13 (about 0.65% of overall test events) results that were too far away from the average. After removing those 13 outliers, the RMSE for those errors was 0.202 seconds and the variance was 0.0401. The scatter plot for these 1,987 errors can be seen in Fig. 13. There were no false negatives, and there were 1,987 true positives.

**Figure 14:**
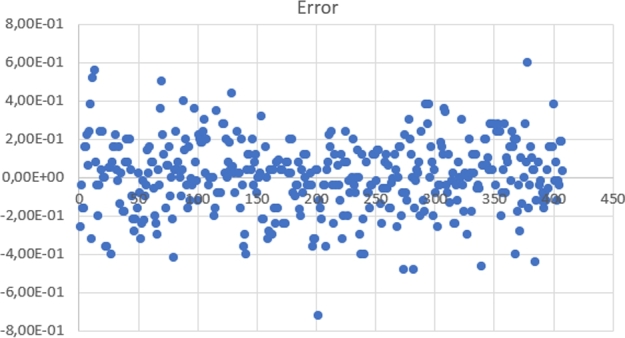
Error time for 422 earthquake events.

Aside from this, there were also 422 new earthquake events, of which 16 events were classified as noise. This means that 16 false-negative events were present. From the 406 earthquake events, there was one result that is too far from the average. After removing the outlier, the RMSE for those errors was 0.1864 seconds and a variance of 0.034. The scatter plot for these 405 errors can be seen in Fig. 14. There were 16 false negatives and 405 true positives. The results show that the overall system performs well on a new dataset.

**Figure 15:**
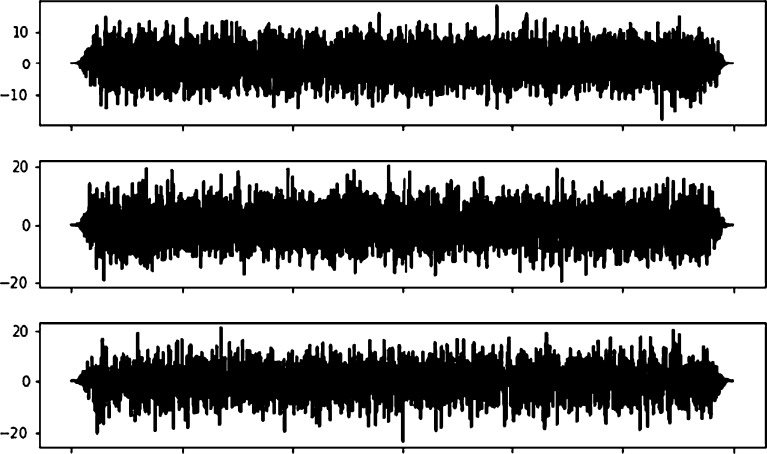
Example of noise event.

Aside from 2,408 earthquake events, 3,559 noise events resemble earthquake signals. Fig. 15 is an example of a noise event.

**Figure 16:**
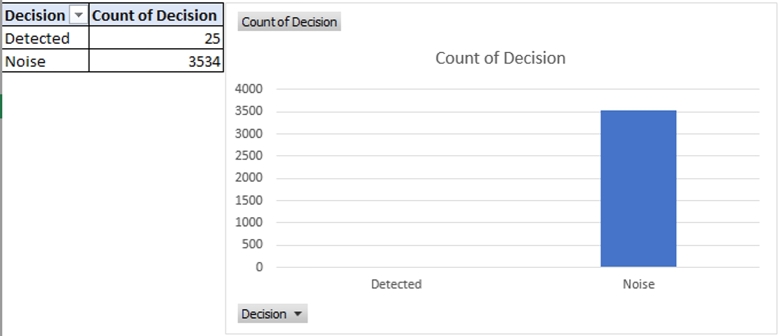
Bar chart for noise events.

When fed with this noise event, the system should consider this noise as a noise, not an earthquake event. The results of the system are shown in Fig. 16.

**Figure 17:**
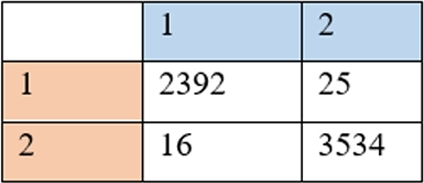
Confusion matrix.

Based on that result, there are 25 false positive present and 3534 true negatives events. Fig. 17 shows the confusion matrix of the overall system. Based on those performances, with the assumption of positive as category 1 and negative as category 2. The row of the confusion matrix represents the system category output, and the column of the confusion matrix represents the target category. It can be concluded that the system had 99.3% accuracy, 99.3% recall, 98.9% precision, and RMSE at 0.202 seconds. It can be said that this system can distinguish noise and earthquake signal well.

Aside from error and accuracy performances, this system can also be considered a candidate for a real-time system. This research tried to implement this system on the i7 processor and Raspberry Pi. On the i7 processor, the execution time for receiving ten windows is 0.17 seconds when detecting a P-Wave on those windows. Meanwhile, the execution time for receiving ten windows is 0.04 seconds when P-Wave is not detected on those windows.

Raspberry Pi has smaller computation resources than the i7 processor. Thus, the model needs to be compressed further for constructing a real-time system. This research uses the lite model prepared by TensorFlow. The lite model decreases the prediction time while maintaining good performance by using a smaller code footprint and less extra parsing when accessing the data. With the lite model, the execution time for receiving ten windows is 0.3 seconds when detecting a P-Wave on those windows. Meanwhile, the execution time for receiving ten windows is 0.1 seconds when not detecting a P-Wave on those windows.

As mentioned above, considering that we used an earthquake signal sampled with 100 Hz and sampled every 0.04 seconds, ten windows mean the periodic time of the AFAP Task is 0.4 seconds. Because the execution time is lower than the periodic time, this system can be scheduled well with a detection speed of 0.4 seconds.

For the record, based on the 1^st^ phase detector window, this system needs 1 second of earthquake signal. This means that the system response for the signal at time t will be at t plus 1 second plus the execution time. There will be no miss-deadline because the execution time is always lower than the periodic time.

## 6 Discussion

Aside from the proposed system configuration, several other configurations were tested. These are shown in Table 5, Table 6.

**Table 5:** Several configurations (1).

Configurations	Accuracy (%)	Precision (%)	Recall (%)	Epochs
1^st^ Phase Detector of the Proposed System	99.33	98.87	99.63	42
1^st^ Phase Detector without Frequency Domain	99.30	98.80	99.60	66
1^st^ Phase Detector without Oversampling	99.43	95.40	89.78	51
1^st^ Phase Detector using SGD	99.17	98.66	99.49	197
2^nd^ Phase Detector of the Proposed System	98.58	98.05	98.75	41
2^nd^ Phase Detector without Frequency Domain	98.20	97.80	98.40	77
2^nd^ Phase Detector without Oversampling	99.39	91.08	76.94	47
2^nd^ Phase Detector using SGD	98.57	98.00	98.80	348

**Table 6:** Several configurations (2).

Configurations	Accuracy (%)	Precision (%)	Recall (%)	Epochs
Phase Picker of the Proposed System	97.50	97.23	97.10	63
Phase Picker without Frequency Domain	96.10	96.40	94.70	75
Phase Picker without Oversampling	99.60	81.30	55.75	32
Phase Picker using SGD	97.00	97.10	96.10	300

The performance of the proposed system is the best of all. Without a frequency domain, all of the performance of the classifiers is decreasing. This is because of the fewer features input for the classifiers.

Without oversampling, the accuracy was good. The precision and recall values are much worse than the proposed system. This is because without oversampling, the training process only uses the undersampling technique, which makes the positive category dataset much smaller than the negative one. That is why recall has a much lower value.

When using SGD, the performance is lower than when using Adam Optimiser. Moreover, it needs more epochs to achieve the same performance as the Adam Optimiser. This would be crucial if the training dataset were much larger.

With the proposed system's overall results, there is a small difference between the predicted and actual arrival times. Note that the accuracy result is not over-fitted but slightly under-fitted. The accuracy performance can be increased with a bigger dataset and more epochs. Another reason for this error is the architecture itself, which has not gone deeper into the features because the proposed system uses only two layers of a convolutional network. Despite that, the accuracy of over 95% and error below 0.3 seconds is sufficient proof to see that this proposed method, an “Image Processing” network, can be used well in this seismic field, and can be developed further. The dataset balancing gives this result with an accuracy of over 95% and an error below 0.3 seconds. Without this method, the training process will not generalise well because of the unbalanced proportion between P-Wave and noise events throughout the training process.

## 7 Conclusion

The proposed system uses frequency and time domains as the input features for the deep learning process by utilising the convolutional and drop-out layers. The result is a system with RMSE at 0.202 seconds and 99.3% accuracy to distinguish between an earthquake and noise. These results imply that the proposed system has succeeded in using an image processing network to solve a seismic problem, namely to detect P-Wave arrival time from an earthquake. For future work, it would be better to use a larger dataset and epoch process for the training to increase its accuracy, decreasing the likelihood of error. In addition, we can try several different architectures to further improve their performance.

## Declarations

### Author contribution statement

Rhesa Aditya: Conceived and designed the experiments; Performed the experiments; Analyzed and interpreted the data; Contributed reagents, materials, analysis tools or data; Wrote the paper.

Carmadi Machbub: Conceived and designed the experiments; Contributed reagents, materials, analysis tools or data; Wrote the paper.

### Funding statement

This research did not receive any specific grant from funding agencies in the public, commercial, or not-for-profit sectors.

### Data availability statement

Data will be made available on request.

### Declaration of interests statement

The authors declare no conflict of interest.

### Additional information

No additional information is available for this paper.
